# Five-year trends in treatment changes in an adult cohort of HIV/AIDS patients in Ghana: a retrospective cohort study

**DOI:** 10.1186/s12879-017-2752-7

**Published:** 2017-10-02

**Authors:** Daniel N. A. Ankrah, Margaret Lartey, Aukje K. Mantel-Teeuwisse, Hubert G. M. Leufkens

**Affiliations:** 1 Korle-Bu Teaching Hospital, P. O. Box 77, Korle-Bu, Accra, Ghana; 2 Division of Pharmacoepidemiology & Clinical Pharmacology, Utrecht Institute for Pharmaceutical Sciences (UIPS), PO Box 80082, 3508 TB Utrecht, the Netherlands; 3 University of Ghana Medical School, P. O. Box GP, 4236 Accra, Ghana

**Keywords:** HIV/aids, Policy change, Database, Utilization, Trends, Ghana

## Abstract

**Background:**

There is limited information on patterns of treatment change among new initiators of highly active antiretroviral therapy (HAART) in the regions most affected by HIV/AIDS. This makes it difficult to identify the determinants of treatment change. In this retrospective cohort study, we examined treatment change patterns over a five-year period among initiators of HAART.

**Methods:**

De-identified data were obtained from the Fevers’ Unit Database at the Korle-Bu Teaching Hospital. All adult treatment-naive patients who started treatment with first line HAART between 1st January, 2008 and 31st December, 2012 were followed over a minimum period of three months. The main outcome was the first treatment change, defined as the first substitution/switch in accordance with the standard treatment guidelines. Data were analyzed stratified by year of treatment initiation. Crude and adjusted hazard ratios were calculated.

**Results:**

A total of 3933 patients were followed with almost equal numbers of initiators per year. The mean age (standard deviation) at treatment initiation was 39 (10.3) years. The most prescribed HAART combination was AZT/3TC/EFV and overall for initiators zidovudine combination therapy was about 60%. Utilization of stavudine containing HAART increased gradually until 2010 and then dropped to zero. Over the study period, 44.9% of recorded deaths were from those initiated with a stavudine backbone, 41.1% from a zidovudine backbone, and 11.5% from a tenofovir backbone. Females had a significantly higher rate of treatment change compared to males (*p*-value = 0.0002), and d4T/3TC/EFV and d4T/3TC/NVP recorded independent treatment change hazard ratios of 12.05 (CI 9.58 to 15.16) and 12.03 (CI 9.27 to 15.61) respectively.. Kaplan-Meier curves showed that treatment change was higher among those who started treatment later in the study period compared with those who started earlier.

**Conclusion:**

A major treatment change in the utilization of antiretroviral medicines in Ghana occurred during the study period which was associated with type of treatment, year of treatment, gender and disease stage. The influence of a policy change during the period may have made a significant impact.. For diseases involving life-long treatment in particular, it is important to monitor and periodically evaluation treatment utilization patterns.

## Background

The most important intervention that delays and prevents the progression of HIV to AIDS, assuming optimum adherence, is treatment with highly active antiretroviral therapy (HAART) [1]. The first drug shown to be effective against HIV was zidovudine; currently there are at least seven classes of HAART in use, mostly in combinations of different antiviral products [2]. This has led to structured treatment strategies, virtually always under the guidance of national or institutional guidelines. Treatment of naïve patients starts with a first line HAART, but regimens may change depending on adverse effects or on inefficacy as a result of development of drug resistance (virological or immunological). Treatment guidelines are adjusted over time based on such insights and evidence. A typical case is the recommendation to phase out stavudine in the management of HIV/AIDS by the WHO from 2010, because of associated mitochondrial toxicities [3–5] manifested as lipodystrophy, lactic acidosis and peripheral neuropathy [6]. This underscores the importance of considering the content and revisions of treatment guidelines when studying time trends of HAART, but such studies are rare in the literature. Notwithstanding its toxicity profile, stavudine is not as expensive as other nucleoside (or nucleotide) transcriptase inhibitors [3], and there are cost-analysis studies showing that it might be cost-effective overall, in some countries [7–9].

In several countries, HIV/AIDS risk populations and treatment cohorts have been studied over the last decades [10–12]. Sub-Saharan Africa has several of such HIV/AIDS cohorts because more than three-quarters of the disease burden is from this region [13]. An example is the KwaZulu-Natal HIV/AIDS cohort nested within the Africa Centre Demographic Information System (ACDIS) cohort [14]. The existence of these cohorts notwithstanding, there is limited information on patterns and determinants of treatment change among new initiators of HAART in the regions most affected by HIV/AIDS.

About 60% of HIV patients in Ghana are female [15]. The HIV prevalence in Ghana routinely conducted by the HIV Sentinel Surveys (HSS) has gradually reduced since 2003. In 2003, 2008, 2013 and 2014, the median HIV prevalence was 3.6%, 2.2% 1.9% and 1.6% respectively [15]. The estimated final transmission rate decreased from 20.3% to 15.9% between 2011 and 2015 [15].

A well-organized government sponsored HIV/AIDS treatment began in the year 2003 following a period of dominance by private retailers who kept no structured records. As of December 2003, HAART for Ghana’s HIV/AIDS population was being managed by only three treatment centers in the country, including the Korle-Bu Teaching Hospital (KBTH) treatment center in Accra. Treatment of HIV/AIDS has since been decentralized, increasing the number of treatment centers from just 3 in 2003 to 197 by December 2014 [15]. The situation has improved over the years but more work is on-going. This study involves the HIV/AIDS cohort at the KBTH. It is a retrospective cohort study that examined HAART initiation and changes per individual inception year over a five-year period (2008–2012).

## Methods

### Study site

The Fevers Unit of the KBTH was the study site. The Fevers Unit is one of many units of the Department of Medicine and Therapeutics. The Unit is responsible for the registration and management of all cases diagnosed as HIV/AIDS at the KBTH, as well as those referred from other health institutions in Ghana. Provision of antiretroviral therapy in the Unit started in December 2003. As of 2015, nearly 10,000 clients have been put on HAART at the treatment site. There are three major out-patient clinic days per week, each with an average clinic attendance of about 120 patients per day.

### Subjects

This was a retrospective cohort study. All treatment naive patients who started treatment with first line HAART between 1st January, 2008 and 31st December, 2012 were eligible for this study if they were 15 years or older, enrolled at the Fevers Unit of KBTH and received HAART at the hospital’s pharmacy. The date of exposure was the first date the patient received HAART and each patient was followed over a minimum period of three months. Patients were followed and analyzed per individual inception year. All patients receiving treatment for prevention of maternal to child transmission (PMTCT) of HIV/AIDS were excluded from the study if they were not on full HAART. PMTCT patients who started on full HAART and those who resumed full HAART were enrolled on the date this occurred.

### Data source

De-identified and anonymous data for this study were obtained from the Fevers Unit's Database at the KBTH. Diagnostic and treatment records of all newly registered HIV/AIDS patients at the KBTH are captured in this database. Variables in this study included patient age, sex, inception treatment type and date of inception, date of next treatment appointment and treatment change, and WHO disease stage at treatment initiation. Reason for treatment change was not captured in the database.

### Study outcome

The main outcome was the first treatment change after inception; each person had at least 3 months of follow-up. First treatment change was defined as the first substitution with another first-line drug or a first switch to a second-line drug as recommended by the treatment guidelines for HAART in Ghana during the study period. In this setting most patients on d4T with good haemoglobin and/or high creatinine levels had their treatment replaced with AZT, while those with acceptable creatinine levels and/or poor haemoglobin levels had a replacement with TDF. Death was defined as any case captured as such in the database. A loss to follow-up was defined as any patient who missed a refill appointment and had no information in the database until 60 days after the next appointment date. For patients who did not change treatment, follow-up was censored at the death date (as captured in database), loss to follow-up, date of transfer to another treatment site, or end of study period, whichever occurred first.

### Data analysis

Frequency distributions of baseline variables were calculated. Age was divided into six categories and treatment initiation and treatment change were captured using the HAART combination. Proportions of those who initiated treatment and those with change were calculated for each type of initial HAART by calendar year. Kaplan-Meier plots of year of treatment initiation and HAART combinations (for nucleoside/nucleotide reverse transcriptase inhibitors (NRTI) and non-nucleoside reverse transcriptase inhibitors (NNRTI) during follow-up were made, and corresponding log-rank test was done for each inception year to compare HAART use patterns for the treatment combinations. Rates of treatment change were calculated for different variables and crude and adjusted hazard ratios and corresponding *p*-values were presented using Cox proportional hazard analysis.

During the study period, HAART guidelines in Ghana were revised two times, the third edition in 2008 and the fourth in 2011 [16]. In the fourth edition, a major amendment was made replacing stavudine (d4T) with an appropriate nucleoside/nucleotide reverse transcriptase inhibitor due to drug-induced side effects of the former. The effects of this event on treatment change was captured in this study. SAS software version 9.3 (SAS Institute, Cary, NC, USA) was used for all analysis.

## Results

Over the 5-year period a total of 3933 patients were followed with almost equal numbers of initiators per year. The mean age (and standard deviation) of females at treatment initiation was 37 (10.0) years and 42 (9.9) years for males, but overall the mean age at treatment initiation was 39 (10.3) years with most patients falling between ages 25 and 54 years (Table 1). Among those patients whose WHO disease stage at baseline was known, WHO stage III was the most predominant group. The most prescribed HAART combination in this study was AZT/3TC/EFV, remaining relatively stable over the years. Overall, zidovudine combination HAART amounted to 60.2% (2366/3933) of all patients initiated on HAART, followed by about 28.4% (1117/3933) for a stavudine combination HAART, and about 10% for a tenofovir combination HAART. Treatment initiation with a stavudine containing HAART increased gradually until 2010 and dropped to zero in 2012. There was an increase in the use of tenofovir (TDF) combination HAART. Table 1 also shows that age group (*p* = 0.0990) and gender (*p* = 0.3995, were not significantly affected with the changing years of treatment..

**Table 1:** Characteristics of five inception cohorts of HAART initiators (2008–2012)

Variable	2008 (%) *N* = 836	2009 (%) *N* = 810	2010 (%) *N* = 804	2011 (%) *N* = 753	2012 (%) *N* = 730	p-value
WHO stage						
I	11 (1.3)	18 (2.2)	108 (13.5)	110 (14.6)	155 (21.2)	<0.0001
II	164 (19.6)	66 (8.2)	133 (16.6)	148 (19.7)	150 (20.6)	
III	105 (12.7)	50 (7.4)	221 (27.5)	343 (45.5)	313 (42.9)	
IV	71 (8.5)	52 (6.4)	123 (15.3)	143 (19.0)	102 (14.0)	
Missing	485 (57.9)	614 (65.8)	218 (27.1)	9 (1.2)	10 (1.4)	
Age group						
≤ 25 years	37 (4.4)	37 (4.6)	35 (4.4)	45 (6.0)	45 (6.2)	0.0990
26–35	253 (30.3)	247 (30.5)	218 (27.1)	205 (27.2)	189 (25.9)	
36–45	265 (31.7)	258 (31.8)	255 (31.8)	246 (32.7)	227 (31.1)	
46–55	154 (18.4)	130 (16.0)	148 (18.4)	131 (17.4)	145 (19.9)	
56–65	37 (4.4)	29 (3.6)	48 (6.0)	30 (4.0)	48 (6.6)	
> 65	90 (10.8)	109 (13.5)	99 (12.3)	96 (12.8)	76 (10.4)	
Gender						
Male	290 (34.7)	301 (37.2)	289 (36.0)	252 (33.5)	241 (33.0)	0.3995
Female	546 (65.3)	509 (62.8)	515 (64.0)	501 (66.5)	489 (70.0)	
Initial treatment						
AZT/3TC/EFV	331 (39.6)	263 (32.5)	271 (33.7)	286 (38.0) 147	317 (42.4)	<0.0001
AZT/3TC/NVP	238 (28.5)	193 (23.8)	166 (20.7)	(19.5)	154 (21.1)	
d4T/3TC/EFV	154 (18.4)	208 (25.7)	239 (29.7)	117 (15.5)	0 (0)	
d4T/3TC/NVP	101 (12.1)	137 (16.9)	103 (12.8)	58 (7.7)	0 (0)	
TDF/3TC/EFV	3 (0.4)	2 (0.3)	7 (0.9)	100 (13.3)	202 (27.7)	
TDF/3TC/NVP	0	1 (0.1)	3 (0.4)	26 (3.5)	41 (5.6)	
OTHER	9 (1.1)	6 (0.7)	15 (1.9)	19 (2.5)	16 (2.2)	
Recorded death						
	32 (3.8)	65 (8.0)	52 (6.5)	44 (5.8)	44 (6.0)	–
Number of treatment changes	**(** ***N*** **= 234)**	**(** ***N*** **= 240)**	**(** ***N*** **= 280)**	**(** ***N*** **= 171)**	**(** ***N*** **= 35)**	
AZT/3TC/EFV	41 (17.5)	19 (7.9)	21 (7.5)	20 (11.7)	16 (45.7)	<0.0001
AZT/3TC/NVP	26 (11.1)	23 (9.6)	13 (4.6)	15 (8.8)	13 (37.1)	
d4T/3TC/EFV	98 (41.9)	118 (49.2)	158 (56.6)	87 (50.9)	0	
d4T/3TC/NVP	66 (28.2)	80 (33.3)	84 (29.9)	40 (23.4)	0	
TDF/3TC/EFV	1 (0.4)	0	1 (0.4)	1 (0.6)	2 (5.7)	
TDF/3TC/NVP	0	0	0	1 (0.6)	2 (5.7)	
OTHER	2 (0.9)	0	3 (1.1)	7 (4.1)	2 (5.7)	
Log-rank test for treatment type						
	<0.0001	<0.0001	<0.0001	<0.0001	0.012	

There was an increasing trend in treatment change with increasing disease severity. Although treatment change among WHO stage II patients was lower than that of WHO stage I patients, this was not significant (p-value = 0.189)

There was no trend among the different age groups and sex regarding treatment changes

**Fig. 1:**
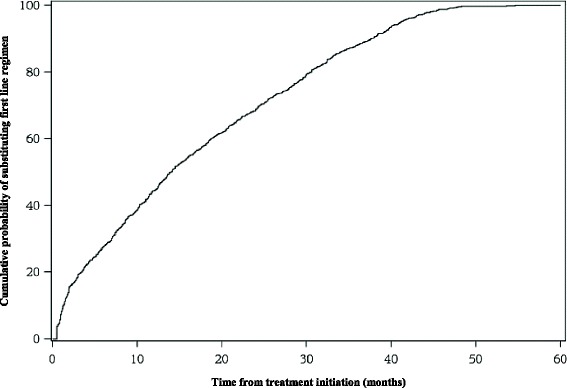
Lifetable of treatment change (in months) over the study period

Recorded deaths (Table 1) as a percentage of those who were initiated HAART from 2008 to 2012 were 3.8 (32/836), 8.0 (65/810), 6.5 (52/804), 5.8 (44/753), and 6.0 (44/730) respectively. Over the study period, 44.9% compared with 41.1% (*p* < 0.0001) of deaths were from those initiated on a stavudine and zidovudine backbone respectfull,, 11.5% from a tenofovir backbone and 2.5% were initiated with other HAART combinations. In Fig. 1 it can be seen that more than 50% of treatment changes occurred before 20 months of follow-up and the last change occurred after 54.5 months of follow-up. We can infer from Table 2 that those who had been on treatment much longer had a reduced rate of treatment change per 1000 person months of follow-up. The highest rates of treatment change were recorded in 2010 (17.9 per 1000 person-months) and 2011 (18.7 per 1000 person-months). In all there were 93.5% substitutions (from one first line to another first line HAART) and 6.5% switches (from a first line to a second line HAART).

**Table 2:** Rates of treatment change, and crude and adjusted hazard ratios

Variable	Enrollees (%)	Treatment changes (%)	Follow-up/months	Rates/ 1000	cHazard Ratio*	95% CI	p-value^∞^	aHazard Ratio#	95% CI	p-value
YEAR	***N*** **= 3933**	***N*** **= 960**								
2008	836 (21.3)	234 (24.4)	30,937.9	7.56	–	–	–	–	–	–
2009	810 (20.6)	240 (25.0)	21,883.7	10.97	1.46	1.21 to 1.75	<0.0001	1.54	1.27 to 1.88	<0.0001
2010	804 (20.4)	280 (29.2)	15,640.9	17.90	2.75	2.27 to 3.33	<0.0001	4.15	3.34 to 5.14	<0.0001
2011	753 (19.1)	171 (17.8)	9137.9	18.73	2.99	2.39 to 3.73	<0.0001	11.55	8.70 to 15.35	<0.0001
2012	730 (18.6)	35 (3.6)	3736.3	9.37	1.33	0.91 to 1.94	0.139	1.43	0.89 to 2.33	0.153
HAART TYPE										
AZT/3TC/EFV	1468 (37.3)	117 (12.2)	33,335.5	3.51	–	–	–	–	–	–
AZT/3TC/NVP	898 (22.8)	90 (9.4)	23,238.4	3.87	1.10	0.83 to 1.44	0.510	1.14	0.85 to 1.53	0.381
d4T/3TC/EFV	718 (18.3)	461 (48.0)	12,751.1	36.15	10.80	8.80 to 13.35	<0.0001	12.05	9.58 to 15.16	<0.0001
d4T/3TC/NVP	399 (10.1)	270 (28.1)	7705.4	35.04	10.42	8.38 to 12.96	<0.0001	12.03	9.27 to 15.61	<0.0001
TDF/3TC/EFV	314 (8.0)	5 (0.5)	2528.6	1.98	0.59	0.24 to 1.45	0.252	0.22	0.09 to 0.56	0.0013
TDF/3TC/NVP	71 (1.8)	3 (0.3)	637.5	4.71	1.43	0.45 to 4.50	0.542	0.58	0.18 to 1.85	0.358
OTHERS	65 (1.7)	14 (1.5)	1130.3	12.39	3.69	2.19 to 6.42	<0.0001	2.99	1.71 to 5.23	0.0001
GENDER										
Male	1373 (34.9)	282 (29.4)	28,625.7	9.85	–	–	–	–	–	–
Female	2560 (65.1)	678 (70.6)	52,701.0	12.87	1.31	1.14 to 1.50	0.0002	1.19	1.01 to 1.39	0.031
AGE GROUP										
< 25	199 (5.1)	44 (4.6)	3573.7	12.31	–	–	–	–	–	–
25–34	1112 (28.3)	277 (28.8)	24,080.0	11.50	0.95	0.69 to 1.31	0.755	1.01	0.73 to 1.40	0.939
35–44	1251 (31.8)	307 (32.0)	25,904.0	11.85	0.97	0.71 to 1.33	0.864	1.03	0.75 to 1.42	0.871
45–54	709 (18.0)	169 (17.6)	14,081.4	12.00	0.99	0.71 to 1.37	0.945	1.09	0.78 to 1.54	0.605
55–64	192 (4.8)	44 (4.6)	3633.0	12.11	0.99	0.65 to 1.51	0.968	0.93	0.60 to 1.42	0.721
≥ 65	470 (12.0)	119 (12.4)	10,054.6	11.84	0.98	0.69 to 1.38	0.903	0.94	0.66 to 1.33	0.706
WHO Stage										
I	402 (10.2)	37 (3.9)	5840.4	6.34	–	–	–	–	–	–
II	662 (16.8)	68 (7.1)	14,568.5	4.67	0.76	0.51 to 1.14	0.189	0.87	0.71 to 1.36	0.294
II	1042 (26.5)	187 (19.4)	15,465.5	12.09	1.93	1.36 to 2.74	0.0003	2.33	1.44 to 3.02	0.007
IV	491 (12.5)	115 (12.0)	7901.6	14.55	2.31	1.60 to 3.35	<0.0001	2.73	1.49 to 4.02	<0.0001
Missing	1336 (34.0)	553 (57.6)	37,550.7	14.73	2.38	1.71 to 3.33	<0.0001	2.83	1.82 to 3.88	<0.0001

*crude hazard ratios; #adjusted hazard ratios for all other variables

Among those on HAART the lowest rates of treatment change was recorded by those on a tenofovir or zidovudine combination therapy. Table 2 shows that d4T/3TC/EFV and d4T/3TC/NVP recorded the highest rates of treatment change of 36.16 per 1000 person months and 35.04 per 1000 person-months respectively, and compared with the reference treatment, these changes were significant (*p* < 0.0001). A tenofovir combination HAART (especially with efavirenz) in this study looked more protective against treatment change compared to the reference HAART (independent *p*-value of 0.013 and 0.358 for TDF/3TC/EFV and TDF/3TC/NVP respectively). Females had a significantly higher rate of treatment change compared to males (p-value = 0.0002). There was no difference in treatment change among different age groups. This is clear from the overlaps of the confidence intervals of the groups. Those with WHO stage IV at baseline had the highest rate of change of treatment (14.55 per 1000 person months).

**Fig. 2:**
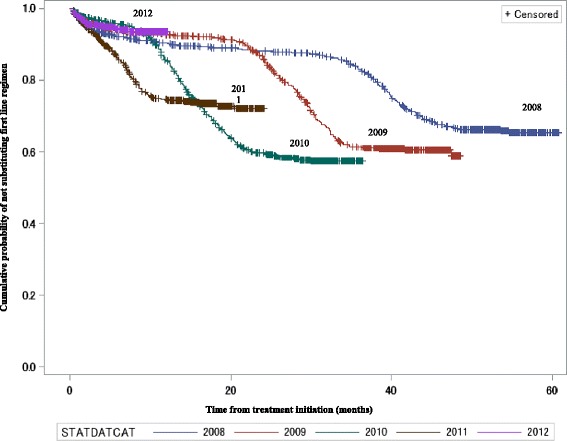
Kaplan-Meier (K-M) plots of treatment change patterns by year from 2008 to 2012

**Fig. 3:**
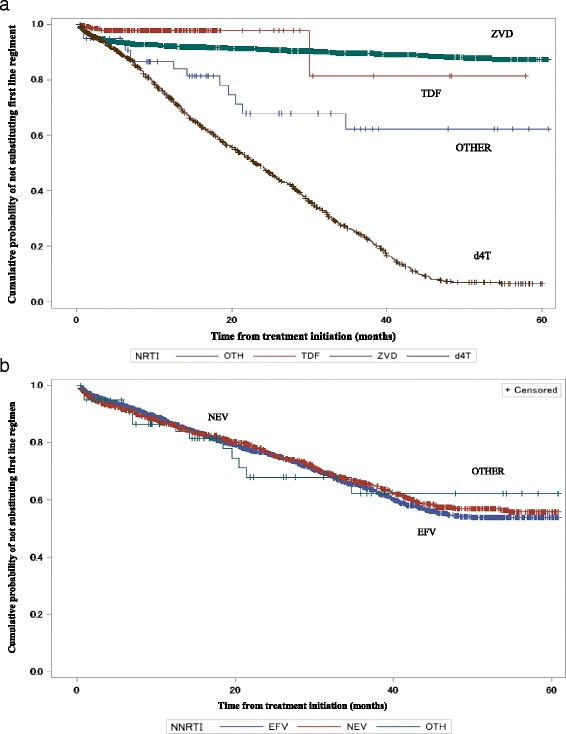
K-M plots of treatment change for the main HAART combinations during follow up. **a** Nucleoside/nucleotide reverse transcriptase inhibitor (NRTI) backbone. **b** Non-nucleoside reverse transcriptase inhibitor (NNRTI) backbone

Kaplan-Meier curves show that treatment change was higher among those who started treatment later in the study period compared to those who started earlier (see Fig. 2). Figure 3A shows that by the end of the study period 91.1%, 97.7%, and 92.0% of those on a zidovudine combination therapy, tenofovir combination therapy, and those on “other” combination therapy respectively, were censored compared with only 34.6% of those on a stavudine combination therapy (*p* < 0.0001); Fig. 3B shows that there was no difference between the two non-nucleoside reverse transcriptase inhibitors (log rank test *p*-value = 0.869). Adjusted hazard rations in Table 2 shows that compared to 2008, changes in 2009, 2010 and 2011 were significantly different, but that of 2012 was not. There was no treatment change difference between the reference age group of less than 25 years and all other age groups in this study. Females had a 19% higher treatment change ratio compared with males.

## Discussion

This study showed that Zidovudine based combination therapy was the most prescribed HAART over time in all the five inception cohorts. By the end of the study period, most patients on stavudine based combination therapy had their treatment replaced with another nucleoside (or nucleotide) reverse transcriptase inhibitor.. Death rates were higher among those initiated on a stavudine combination backbone compared to those on zidovudine or tenofovir combinations. Independent hazard ratios showed that apart from age group, all other variables had a role to play concerning treatment change in this study.

Death rates in this study were significantly higher among those on stavudine compared to zidovudine. In a study by Laurent et al. in Cameroun using ZVD/3TC/NVP and d4T/3TC/NVP [17], a similar observation was made. Most antiretroviral medicines are associated with documented side effects, hence the need for continuous monitoring. Although this study did not look at risks of HAART, it is recommended that databases should capture such occurrences to promote further studies. The low number of switches in this study compared to substitutions show that most of the treatment changes were not as a result of treatment failure. In a previous study on treatment change at the same study site during the period 2004 to 2009, Ankrah et al. [18] observed that treatment switching accounted for about 20% of the changes compared to just over 6% in this study.

In the first three years during follow-up treatment change was initially gradual. This was followed by a period of increased treatment change and then it ended with another gradual phase. Those who started treatment from 2011 missed the initial gradual phase but experienced a steep phase followed by a gradual treatment changing phase. This implies that something may have triggered the treatment change after 2010. In particular, in the 2010 revision of the antiretroviral therapy for HIV infection for adults and adolescents [19], the WHO made a general recommendation of a progressive replacement of stavudine in HIV/AIDS treatment centers, and gave directions on how less endowed health systems should roll out these changes in order to avoid wastage and contain cost. The need to change was as a result of empirical evidence of toxicity associated with the use of stavudine [3–6, 20, 21]. The actual change however, depended on how such countries adopted and adapted to the recommendations. In Ghana patients suspected to be at increased risk of toxicity were the first to be affected, and gradually, all those on stavudine had their treatment replaced with either a first or a second line treatment depending on the presenting situation.

It was evident that most patients in this cohort, originally on stavudine, had a replacement with tenofovir over time. These changes were made based on well informed reasons. Stavudine-related dyslipidemia was reduced significantly after replacement with tenofovir [22]. This was due to improvement in total cholesterol, low density lipoprotein cholesterol and triglyceride levels [22]. In a randomized double blind study, Gallant et al. [23] reported that tenofovir was as efficacious as stavudine but the former had better lipid profiles and reduced lipodystrophy compared to the latter. Results from this study show that a tenofovir combination HAART (especially a combination with efavirenz) may have a slight edge over a zidovudine combination HAART. In a systematic review and meta-analysis, a tenofovir combination HAART showed superior viral load suppression, and was better tolerated by patients [24]. However, emtricitabine, instead of lamivudine was the supporting drug. Normally tenofovir combination HAART is given as a single daily dose compared to a twice daily dose for zidovudine. This may lead to better adherence with a tenofovir HAART. Furthermore, tenofovir is the drug of choice among HIV patients co-infected with hepatitis. While tenofovir can easily replace zidovudine if there is a toxicity among patients on the latter drug, the vice-versa cannot be easily done where patients co-infected with hepatitis are concerned. All that said, the use of tenofovir in this setting is not as established as that of zidovudine. It will take us some time to make a final decision on which combination is the better of the two medicines. Changes from stavudine to zidovudine as observed in this study were moderate, in line with what we know about the differential profiles of these products [25]. These activities form part of the commitment of HIV policy makers and health care workers to ensure that HIV/AIDS patients attain optimum quality of life during treatment.

Our results underscore how timely the Ghanaian health system adhered to WHO’s new treatment policy intervention. This was similar to the situation in Botswana [26] where the authorities did not wait for stavudine associated signs and symptoms to occur before considering to change. Approaches of this nature have been described as very good [27] because of the progressive nature of stavudine related symptoms [28]. Data capture also improved during the period. This was particularly reflective by the fact that in the 2008 cohort almost two-thirds of the cohort had no recording of WHO disease stage, however, in the 2012 cohort WHO disease classification was almost complete.

The observation of low frequencies of deaths in this study may be due to various reasons. These include our inability to determine the causes of loss to follow-up (discharges, abscondees, unreported deaths) from the database. It may also be due to conscientious treatment offered at the treatment center. This is because the study site is a national referral site with an increased probability of having the sickest HIV/AIDS patients in the country. The large numbers of missing values, for example, data on WHO stage was a limiting factor that calls for further reinforcement in the data capture at the treatment site. Another limitation was the parsimonious nature of the variables in our dataset. This may have led to a number of unmeasured confounders, the presence of which may have better explained the reasons for treatment change in this study.

## Conclusion

This study has shown that between 2008 and 2012, utilization of antiretroviral medicines in Ghana went through a major transition. In this period, stavudine was replaced with tenofovir, another nucleoside (or nucleotide) reverse transcriptase inhibitor. Stavudine has since been phased out of HIV/AIDS treatment among adults in Ghana. This study shows that these changes were as a result of several factors including the type of treatment, year of treatment, gender and WHO disease stage. International policy changes firmly grounded on empirical evidence may have also contributed to this study’s findings.. During the period HIV/AIDS data capture also showed some improvement. For diseases involving life-long treatment, it is important to monitor and periodically evaluate treatment utilization.

## Abbreviations

3TC: Lamivudine
ACDIS: Africa Center for Demographic Information Systems
AIDS: acquired immunity deficiency syndrome
AZT: Zidovudine
EFV: Efavirenz
HAART: highly active antiretroviral therapy
HIV: human immune virus
KBTH: Korle-bu. Teaching Hospital
PMTCT: prevention of maternal to child transmission
TDF: Tenofovir
WHO: World Health Organization
